# Retraction Note: Investigation of the hub genes and related mechanism in ovarian cancer via bioinformatics analysis

**DOI:** 10.1186/s13048-015-0147-1

**Published:** 2015-03-26

**Authors:** Ling-jie Fu, Bing Wang

**Affiliations:** Department of gynaecology and obstetrics, Shengjing hospital of China Medical University, No.36 Sanhao Street, Shenyang City, 110004 China; Ultrasound Department, Shengjing hospital of China Medical University, No.36 Sanhao Street, Shenyang City, 110004 China

## Retraction

The Publisher and Editor regretfully retract this article [1] because the peer-review process was inappropriately influenced and compromised. As a result, the scientific integrity of the article cannot be guaranteed. A systematic and detailed investigation suggests that a third party was involved in supplying fabricated details of potential peer reviewers for a large number of manuscripts submitted to different journals. In accordance with recommendations from COPE we have retracted all affected published articles, including this one. It was not possible to determine beyond doubt that the authors of this particular article were aware of any third party attempts to manipulate peer review of their manuscript.
